# Induced phase transformation in ionizable colloidal nanoparticles

**DOI:** 10.1140/epje/s10189-023-00386-4

**Published:** 2023-12-07

**Authors:** Leticia López-Flores, Monica Olvera de la Cruz

**Affiliations:** 1 Department of Materials Science and Engineering, Northwestern University, Evanston, IL 60208 USA; 2 Department of Chemistry, Northwestern University, Evanston, IL 60208 USA; 3 Department of Physics and Astronomy, Northwestern University, Evanston, IL 60208 USA

## Abstract

**Abstract:**

Acid–base equilibria directly influence the functionality and behavior of particles in a system. Due to the ionizing effects of acid–base functional groups, particles will undergo charge exchange. The degree of ionization and their intermolecular and electrostatic interactions are controlled by varying the pH and salt concentration of the solution in a system. Although the pH can be tuned in experiments, it is hard to model this effect using simulations or theoretical approaches. This is due to the difficulty in treating charge regulation and capturing the cooperative effects in a colloidal suspension with Coulombic interaction. In this work, we analyze a suspension of ionizable colloidal particles via molecular dynamics (MD) simulations, along with Monte Carlo simulations for charge regulation (MC-CR) and derive a phase diagram of the system as a function of pH. It is observed that as pH increases, particles functionalized with acid groups change their arrangement from face-centered cubic (FCC) packing to a disordered state. We attribute these transitions to an increase in the degree of charge polydispersity arising from an increase in pH. Our work shows that charge regulation leads to amorphous solids in colloids when the mean nanoparticle charge is sufficiently high.

**Graphical abstract:**

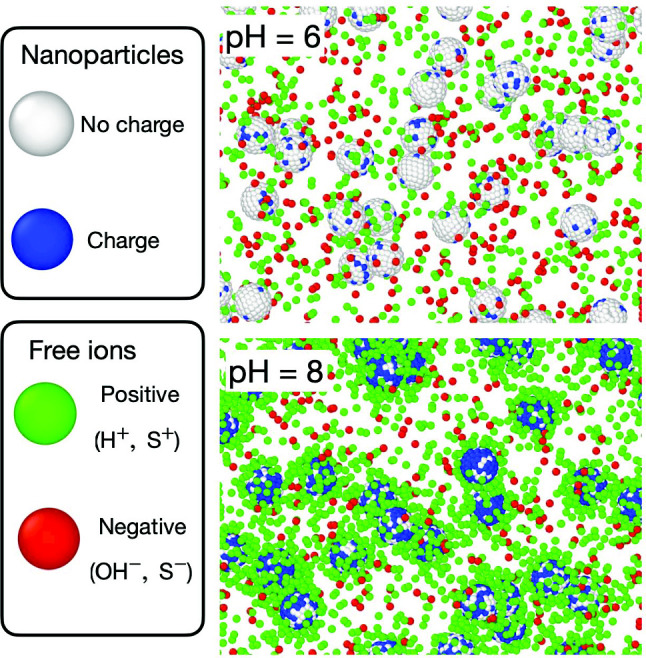

## Introduction

Ionizable surface functional groups are observed in biology and soft materials [1]. These groups can release or combine with a hydrogen ion, and in this way acquire or lose charge, respectively [2]. Ionizable functional groups exist in numerous systems, such as polyelectrolytes, micelles, fibers, proteins, membranes, and colloids [3–13]. An important property of these systems is their responsiveness to an increasing pH. An illustrative example of this phenomenon involves the reaction-driven release of ions from the surface of the particles into the surrounding solution [14, 15]. This process imparts cooperativity in the distribution of surface charge on the particles. That is, the charge on the particles modify the electrostatic interactions, which, in turn, modify the particles charges. This recursive relation leads to the emergence of different states, such as glasses. Herein, we analyze how the charge distribution in a colloid suspension evolves and adapts to new chemical environments.

The process of charging and discharging an acid or base is referred to as *charge regulation* (CR) [16, 17]. Charge regulation is a mechanism that maintains chemical equilibrium in complex systems in response to changes in pH, temperature, and ionic strength. Different approaches have been used to describe CR effects including theoretical, [18] and more recently, computer simulations [19–22]. Moreover, these studies offer insights into the interplay between charge distribution and the behavior of charged entities in complex systems. Because of the practical implications in fields like nanotechnology, engineering, and pharmaceuticals, elucidating the phenomena that can control CR is of utmost importance.

The intersection of charge regulation and structural transitions introduces a challenge when electrostatic interactions play a central role. The CR effect reflects the adjustment of charge on pH responsive groups due to specific chemical reactions. This process profoundly influences the interaction of particles and molecules in a system, and significantly impacts the final structure. In particular, the readjustment of charge in a system can gradually slow the movement of particles, leading to a diverse landscape of structures that the system can access, such as crystalline or amorphous solids. Moreover, studying the interplay between CR and phase transitions presents a challenge due to the combination of chemical reactions and electrostatic interactions arising in these systems.

The objective of this paper is to elucidate the phase behavior in response to pH changes in a system of surface functionalized nanoparticles with acid–base groups. Our model is analogous to nanoparticles or micelles with surface ionizable groups that can be observed in nature. The pH causes a change in the charge distribution on the surface of the nanoparticle, which imparts charge polydispersity leading to different structures such as disordered, crystalline or fluid phases. To construct the phase diagram, we study the structural and dynamic properties as a function of pH by varying the density of nanoparticles in the system.

## Methodological aspects

**Fig. 1:**
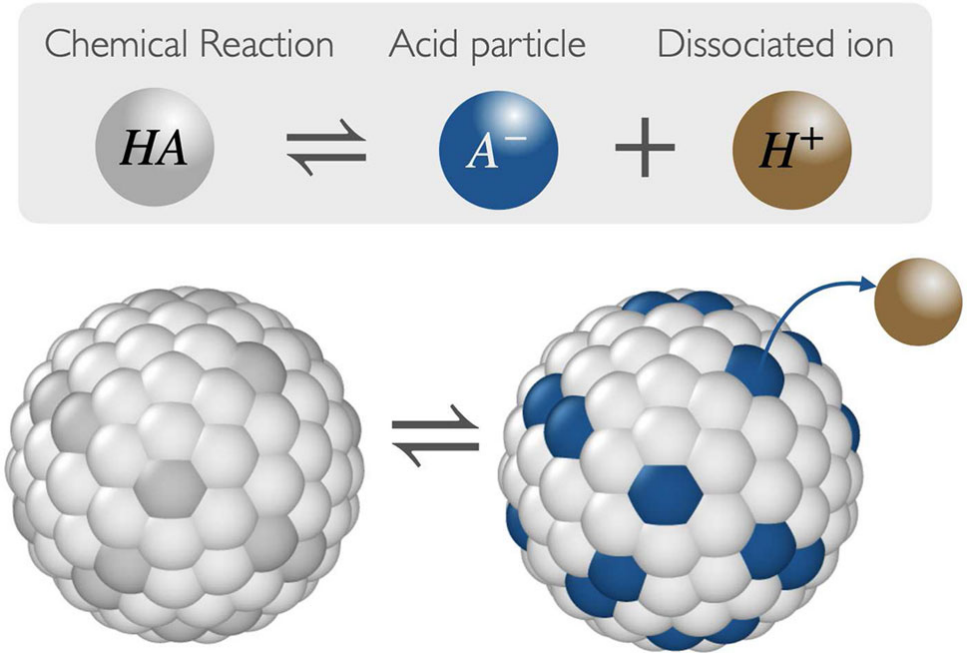
Schematic model for a spherical nanoparticle under a chemical reaction $A \rightleftharpoons A^{-} + H^{+}$. The spherical particle carries the acid particles and delivers the dissociate ions to the solvent

We study the CR effect between negatively charged colloidal nanoparticles of diameter $\sigma _b=5l_B$, immersed in a uniform implicit solvent of dielectric constant $\epsilon$ and temperature *T*, where $l_B=e^2/(4\pi \epsilon _0 \epsilon k_B T)$ is the Bjerrum length, *e* is the elementary charge, $k_B$ is the Boltzmann constant, and $\epsilon _0$ is the vacuum permittivity. Each nanoparticle contains $n_A$ weak acid groups carrying zero charge (neutral state *A*) or an elementary charge *e* (dissociated state $A^-$) and is accompanied with dissociated ions ($H^+$, $OH^-$). The acid dissociation reaction is given by

1 $$\begin{aligned} A =A^- + H^+ \end{aligned}$$

with equilibrium dissociation constant *pKa*. Figure 1 represents a schematic model illustrating a nanoparticle undergoing the chemical reaction given by Eq. 1. Upon the dissociation of a functional group, the particle situated on the surface of the nanoparticle acquires a negative charge, while a positive charge is realized into the surrounding medium as a free ion.

The nanoparticles are immersed in an aqueous solution where $l_B=0.7$ nm, with typical pH and monovalent salt concentration values. We set $pI_{S^{\pm }}=2$, which corresponds to 10 mM of salt; $n_A=110$ acid dissociation groups are represented by small spheres of diameter $\sigma =l_B$, and they are randomly distributed on the nanoparticle’s surface with a $pK_a=6.5$. The total number density of the nanoparticles, $\rho$, is related to the volume fraction, $\phi =\pi \rho \sigma _c /6$. The excluded volume interaction is modeled by the Lennard–Jones (LJ) potential

2 $$\begin{aligned} {u_{ij}(r)=4\epsilon \Big [\Big ( \frac{\sigma }{r}\Big )^{12}-\Big ( \frac{\sigma }{r}\Big )^6+\frac{1}{4} \Big ], \quad r \le 2^{1/6}\sigma } \end{aligned}$$

where $\sigma$ is the diameter of the small particles. The repulsion strength is set to $\epsilon =k_BT$, which provide a sufficiently strong repulsion to avoid the overlap of the particles.

The nanoparticles interact via LJ and Coulomb potentials. We use the particle-particle-particle-mesh (PPPM) algorithm with a relative precision of $10^{-4}$, to solve the long-range electrostatic interactions. To compute the charge regulation effect, we use the methodology described in Ref. [22]. The temperature is controlled by a Langevin thermostat with a damping factor equal to 20$\tau$, where the unit time $\tau$ is based on the mass *m* of the ions and dissociable groups. After every 400 MD steps, we perform 200 MC steps for the charge regulation. When the system is diluted, the time of equilibration is longer than when we have a concentrated system. Following equilibration, we obtain the particle charge average as a function of the pH for different concentrations of nanoparticles with fixed salt concentration. At low concentration, we recover the behavior reported in Ref. [13] and the charge of one particle obtained using the Poisson-Boltzmann equation [23]. The results are expressed in LJ units, where *M*, $\sigma$ and $\epsilon$ are the units of mass, length and energy, respectively. The time unit is $\tau =\sqrt{M\sigma ^2/k_B T}$. The simulations were conducted with $N=108$ nanoparticles formed by $n_A$ small particles in a cubic simulation box with periodic boundary conditions. The initial configuration was placed on an FCC lattice at the desired density. We run the simulation for $10^5$ time steps, and the interaction between particles is only given by excluded volume interactions. This procedure is performed to generate a random configuration for all systems. Once the initial configuration is constructed, several thousand cycles are performed to allow the system to equilibrate with respect to CR of the nanoparticles and the addition of the ions and dissociable groups. This is followed by at least two million cycles and the data is collected every hundred cycles. The simulation starts with an electrically neutral system, where the particle is uncharged. We compare a system with uncharged nanoparticles and a system with charged nanoparticles when subjected to different pH environments and find that both converge to similar charge distributions. Throughout the simulation, pair of ions are inserted or deleted to maintain the electroneutrality. To compute an average charge distribution on the nanoparticles in our system, we average over 100 different configurations.

The structural and dynamic properties are calculated from the equilibrium configurations generated in the simulations. We follow the center of mass trajectory of each nanoparticle to calculate the properties reported in this paper. Moreover, the radial distribution function was obtained by using the standard approach [24].

The mean squared displacement (MSD) is calculated to follow the transition from the liquid to a solid state. The MSD is given by

3 $$\begin{aligned} W(t)=\langle [\Delta \vec {r}(t)]^2\rangle / 6 \end{aligned}$$

where $\Delta \vec {r_j}(t)=\vec {r_j}(t)-\vec {r_j}(0)$. In the limit of long time, the MSD reads $W(t)/\sigma ^2 \approx D_L^* t/\tau$, which is proportional to the scaled long-time self-diffusion coefficient $D_L^*\equiv D_L/D^0$.

## pH-dependent phase transitions of systems with ionizable groups

In this section, we analyze the simulation results for negatively charged ionizable groups on the surface of the nanoparticles as a function of pH. For this, we consider a liquid formed by $N=108$ nanoparticles in a volume V, interacting through an excluded volume LJ potential, given by Eq. (2), where the diameter is given by the center of mass of the nanoparticle, i.e., $\sigma _b$. In addition, Coulomb interactions between all charges are computed. The state space of the system is spanned by two variables: the number density $n=N/V$ and the pH. For fixed temperature $T=1$, the equilibrium phase diagram of the system is provided in the space (pH, $\phi$), which contains the crystalline and amorphous solid phases.

A crystalline transition occurs as the density of the particles increases; the system undergoes a transition from a fluid-like state to an ordered crystalline state. This transition is characterized by the sudden appearance of long-range positional order, where particles occupy specific positions within a repeating lattice. Unlike crystallization, which involves the formation of an ordered lattice, the glass transition leads to the formation of a disordered state, which does not have long-range translational order, hereafter referred to as an amorphous solid.

**Fig. 2:**
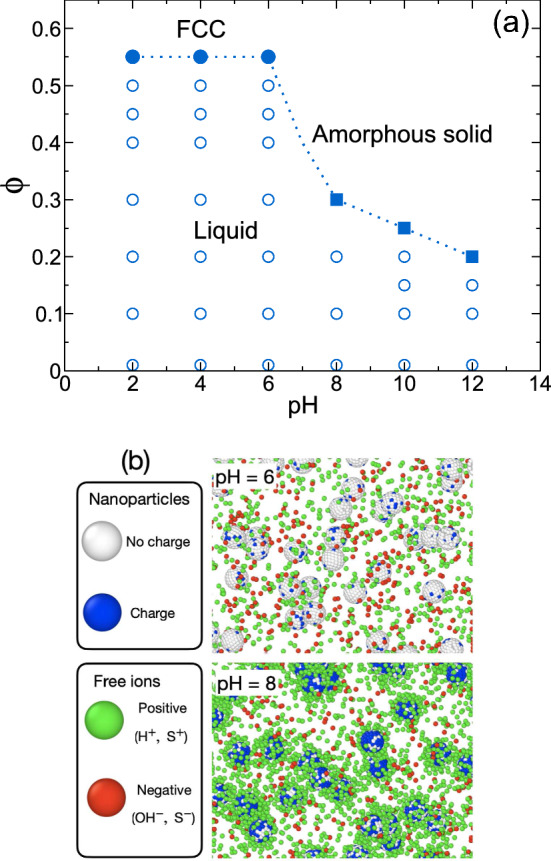
State space of the system spanned by the variables ($\phi$, pH). **a** The open circles imply the system is in a liquid state, and the full symbols imply the system has transformed into a different structure. For a pH between 2–6, the system is in a FCC structure. For a pH between 7–12, the structure is an amorphous solid. **b** Snapshots of characteristic structures of the simulation at $\phi =0.01$ and two different values of pH

From the analysis of structural and long-time asymptotic properties, we show in Fig. 2 the liquid-crystalline-glass phase diagram as a function of pH. At low pH (2, 4, 6), we observe that when the density of colloidal nanoparticles is increased, the system forms an FCC crystalline structure. This phenomenon occurs when the volume fraction $\phi \simeq 0.55$. Nevertheless, if the pH exceeds 6, we observe that the FCC structure is destroyed, and we get a disordered structure. To characterize these phases in our system, we analyzed the average charge of the particles, the configuration of the systems, and the static and dynamic properties. The analysis of all the phase transitions are presented below. Furthermore, the simulation shows that the nanoparticles are entirely discharged at low pH (pH 2), exhibiting only hard-sphere behavior. However, when pH is increased to 4–6, we find that some surface groups are ionized, which allows us to get charged colloidal nanoparticles. In Fig. 2b, the charge regulation configurations of the system at a volume fraction of $\phi =0.01$ are presented for two distinct pH levels. When the chemical reaction occurs, the nanoparticles can acquire two states: a discharged state (white small particle) and a charged state (blue small particle). The nanoparticles are surrounded by cations (green particles) and anions (red particles). The cations include the release particle ($H^+$) and the salt cations ($S^+$) and the anions include the hydroxyl ($OH^-$) and the salt anions ($S^-$). We note that in Ref. [13], the attraction of the nanoparticles is equal to $l_B/\sigma =\lambda / k_B T =64$, which represents a low dielectric media. In our case, we have the electrostatic interaction at the contact $l_B/\sigma =\lambda / k_B T =1$, corresponding to a model for NaCl dissolved in water at room temperature. Due to these differences, the structures in our systems are completely different than those reported in Ref. [13].

**Fig. 3:**
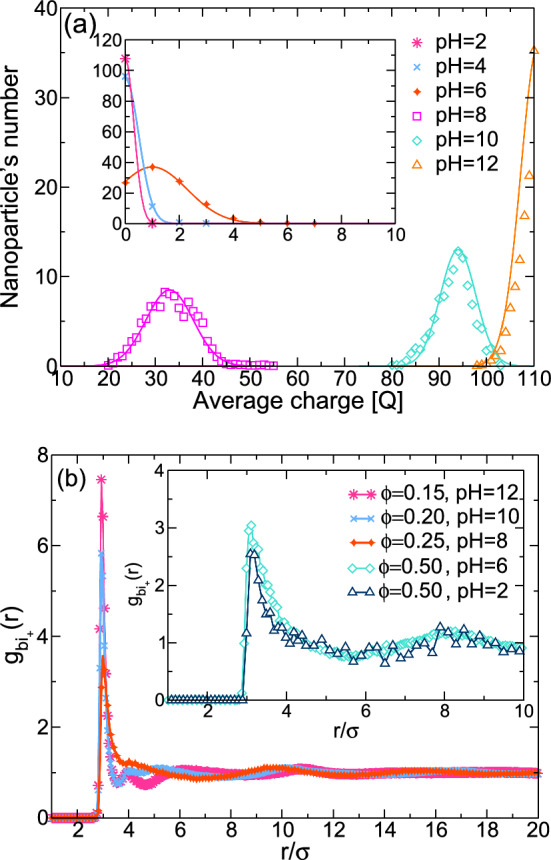
Average surface charge on the nanoparticle as a function of the number of total nanoparticles for different values of pH. The line represents a fitted Gaussian distribution given by the equation $f(x)=ae^{-c(x-b)^2}$. Average structure $g_{bi_+}(r)$ between the center of mass of the nanoparticle and the positive ions at different volume fraction and pH values

In Fig. 3, we show the average surface charge (Q) with respect to the distribution of nanoparticle number at different pH values. The average charge is obtained at the phase boundary of Fig. 2. These curves are histogram of number of particles of a given charge. In each distribution (nearly Gaussian), the total sum of particles corresponds to the overall number of nanoparticles within the system. At pH 2 and 4, we find that each nanoparticle contains zero or a few charges on average on the surface when the system is in equilibrium. As an illustrative example, at pH 4, the system contains 98 discharged nanoparticles and 10 particles with one charge on average. This result implies a narrow Gaussian distribution of charge on the surface of the nanoparticles. When pH is increased to 6, we find that the average charge is normally distributed. However, the average charge of each nanoparticle in the system is around one ionizable ion on each particle, as shown in the inset of Fig. 3. This average charge is insufficient to break the FCC structure. To get disordered states, pH must be increased, the increase in the average charge on the nanoparticles. In the main panel of Fig. 3, we observe the average charge is a Gaussian distribution, and the average charge increases as a function of pH. The increase in the average charge facilitates the accumulation of ions around each nanoparticle. Figure 3b shows the distribution of ions around the nanoparticles. Notably, at pH levels around 2–6, the ions exhibit hard-sphere liquid behavior, due to the absence of interaction between the nanoparticle and the small ions. However, as the pH increases, the primary peak of $g_{bi_+}(r)$ increase, accompanied by the emergence of a secondary peak at $r=4$. This outcome aligns with the observations presented in the snapshots of the simulation illustrated in Fig. 2b, wherein an increase in pH corresponds to the presence of positive ions surrounding the nanoparticles. The excluded volume fluctuates in the simulations due to dissociation, where ions are added to maintain electroneutrality. As the pH increases toward the transition point, the number of positive ions increases due to the dissociation of charges on the nanoparticles, raising the exclude volume. Therefore, the free ions play an important role in the driving force behind the transitions

**Fig. 4:**
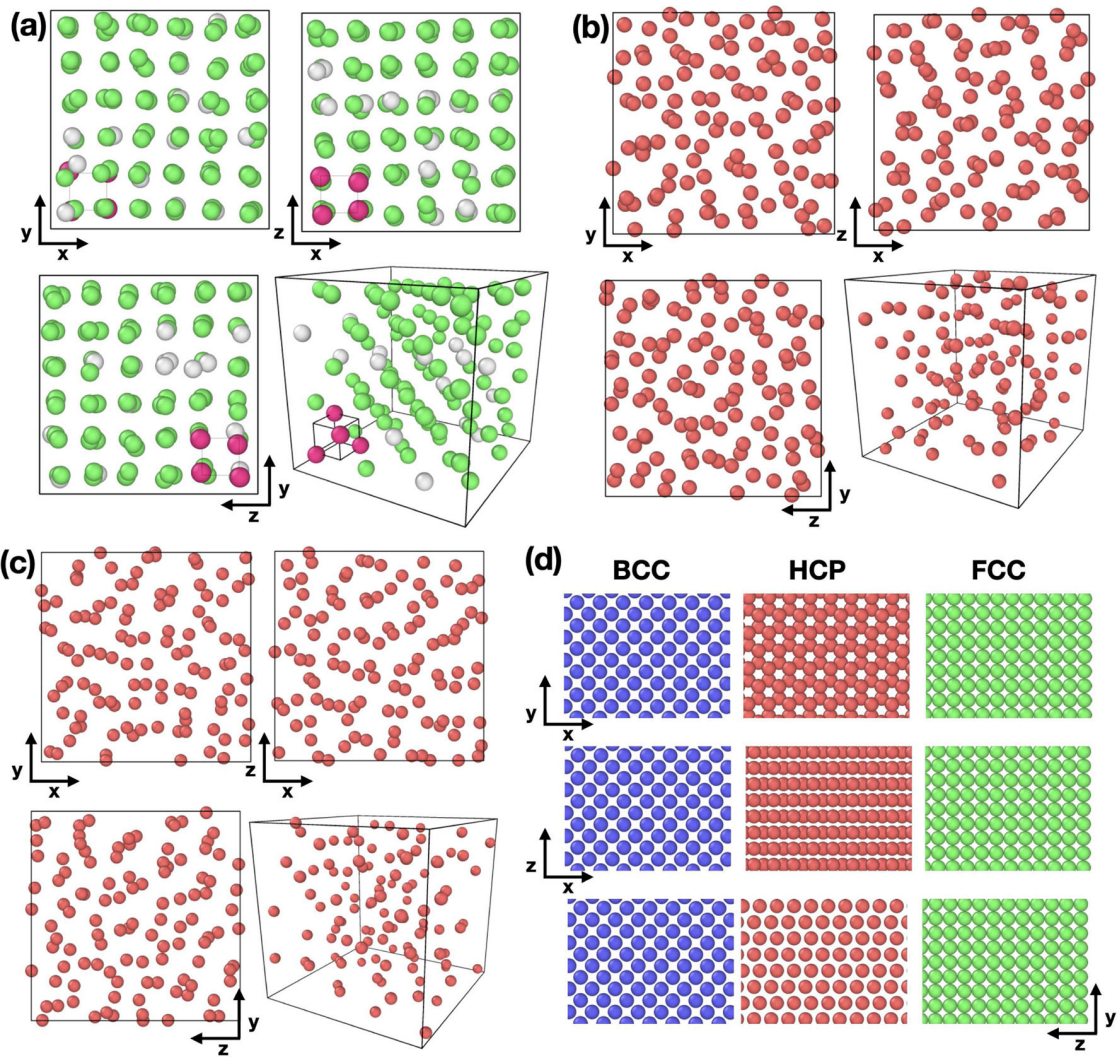
Configuration of the center of mass of the nanoparticles at **a** (pH,$\phi$)=(2,0.55), **b** (pH,$\phi$)=(8,0.30), and **c** (pH,$\phi$)=(12,0.20). We show different perspectives of the system to determine the structure in the system. The magenta particles in (**a**), attached to the small cube represent the FCC unit cell. **d** Configuration of the ideal crystalline structure ($T=0$) in the planes x–y, x–z and z–y for reference. The magenta particles represent the positions (0,0,0),(0,0.5$\sigma _b$,0.5$\sigma _b$), (0.5$\sigma _b$,0,0.5$\sigma _b$) and (0.5$\sigma _b$,0.5$\sigma _b$,0) which corresponds to the FCC unit cell coordinates of the particles

In Fig. 4a, we show the center of mass (CM) of the colloidal nanoparticle at pH 2. The configuration shows the CM of the nanoparticle maintained in an FCC structure, and the CM is around each order position in the structure. The phase diagram in Fig. 2 shows that as pH exceeds 6, we observe a density decay. This decay is due to the presence of electrostatic interactions in the system. As mentioned previously, the surface charge of the nanoparticles increases as a function of the pH. This increase in surface charge enhances the repulsion between particles as the density decreases. These configurations are in Fig. 4b and c where we can observe the CM of the nanoparticles in a disordered state. In our work, we recover previous results in the low and high pH limits. At low pH, the dominating contribution is the hard-sphere term. In this scenario, where polydispersity is absent, our findings consistently lead to the formation of the face-centered cubic (FCC) structure [25, 26]. Conversely, when the nanoparticles are negatively charged and certain types of polydispersity are introduced, such as variations in nanoparticle size, previous work has shown the emergence of a disordered state [27]. Additionally, Fig. 4d shows three different ideal crystalline structures in different planes: body-centered cubic (BCC), hexagonal close-packed (HCP) and face-centered cubic (FCC). The snapshots of FCC on the planes in Fig. 2a have similar configuration to the FCC ideal structure. The difference in our snapshot obtained from the simulation is that the particles are outside the ideal structure. The Landau-type analysis of Alexander and McTague [28] argues that weekly first order transitions from the liquid state to an order solid should be to the body-centered cubic (BCC) phase. In our system, at low pH, the main contribution is the hard-sphere term. In this limit, experiments and Monte Carlo simulations involving hard spheres have consistently pointed to the face-centered cubic (FCC) phase as the most stable configuration [25, 26, 29, 30], in agreement with our results at low pH. However, in the case of the system involving charged colloids, experimental observations have validated that under specific conditions, such as in low salt concentration (typically less than 0.002 mM) or low volume fractions (usually less than 0.008), the BCC structure is the one that is encountered upon going from the liquid state to an ordered solid [31, 32]. For our system, when we increase the charge, due the polydispersity, we have disordered states. The analysis of the static and dynamic properties provides information about the transition of the systems.

**Fig. 5:**
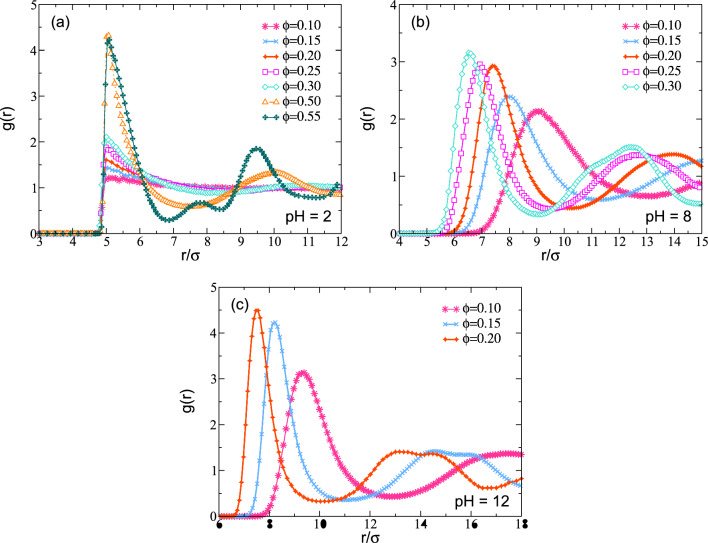
Radial distribution function of the center of mass of the nanoparticles at different values of $\phi$. The figure **a** provide the information of the system at pH 2, **b** at pH 8 and **c** pH 12

**Fig. 6:**
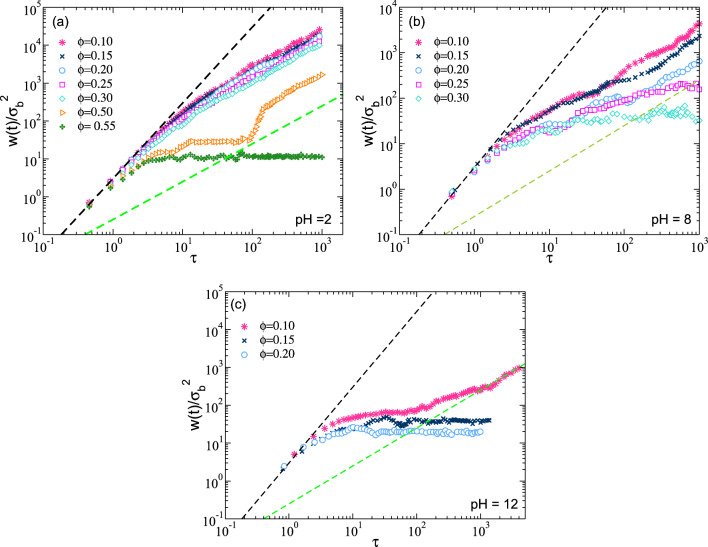
Mean square displacement for macromolecules at different volume fractions. The figure shows the MSD for different values of pH. These values are **a** pH 2, **b** pH 8 and **c** pH 12. The dark dashed line represents the quadratic behavior given by the expression $f(t)=3t^2$, and the green dashed line represents the linear behavior given by $f(t)=0.25t$

In the case of the static structure, we obtain the radial distribution function *g*(*r*) at different concentrations and pH values. In Fig. 5, we plot simulation results for *g*(*r*) of the CM of the nanoparticles at different pH. In Fig. 5a, we show the result for pH 2, where at high densities $\phi =0.55$, we get an FCC structure. At low volume fraction $\phi$, we observe that *g*(*r*) is a flat line around the value 1. This means the particles do not interact between each other, i.e., the only interaction between them is excluded volume. When the density is increased, we observe that *g*(*r*) oscillates around 1, which means the particles are correlated. Also, at $\phi =0.55$, we find the second peak at $r=7.5\sigma$, which means that the first and second nearest neighbors are at one distance equal to 1.5. If we have an FCC structure in a unit cell, the distance between the particle from its nearest neighbors is when $d=\sqrt{2}\sigma =1.41\sigma$, which is close to the distance we get for the average of the *g*(*r*). This information corroborates the result of the system is an FCC structure, obtained in Fig. 4b. However, if we change the pH to a higher value of 8, we observe that the peak around $r=7.5$ disappears, and the second peak now occurs at bigger distances. Additionally, the oscillations of *g*(*r*) are more pronounced at lower volume fractions when compared with results obtained for pH between 2–6. This means the electrostatic interaction plays an important role when the pH exceeds 6. In this case, we need to analyze the dynamic properties to get information on if the system is in a liquid state or is in some glassy state.

In Fig. 6, we show the MSD, following the center of mass of the nanoparticles, scale in reduced units, i.e., $W(t)/\sigma _b^2$, which is a function of time scaled $t/\tau$. As we observe, the results display the ballistic behavior (at short times) [$W(t)\approx 3v_0^2t^2$] and a diffusive regime (at long times) [$W(t)\approx D_L t$] at different values of pH. Furthermore, we observe that at low pH, the MSD has a flat behavior at $\phi =0.55$. This means that the particles are localized for a long time. However, when we have a disordered system, we observe a flat behavior when we increase the volume fraction. This is a well-known effect when we have disordered states or glasses [33–35]. That is, a particle is caged by the neighbors, demonstrating a glass phase [36, 37]. Using all these elements, we characterize the phase diagram as a function of the pH for a system with ionizable groups on the nanoparticles.

## Conclusion

We analyze charge regulation effects on colloidal solutions of nanoparticles that contain acid-ionizable groups on their surface via simulations. The simulations include Coulomb interactions and hard-core repulsions. Charge regulation is an entropic effect that affects the charge of the particles, and the Coulomb potential energy affects their interactions, which, in turn, modify the charge on the nanoparticles. The phase diagram as a function of the pH is obtained by analyzing the structure and dynamical properties, as well as the configurations of the equilibrium states. The phase diagram shows a liquid state at any pH value when the volume fraction of nanoparticles is low. When the pH value is low and the nanoparticles have low or nearly zero charge, a crystalline structure emerges at large nanoparticle volume fractions. However, at large pH values such that the electrostatic interactions are significant, a disordered structure arises at intermedium nanoparticle volume fractions. We find that the disordered states have a large degree of charge polydispersity; that is, when the average nanoparticle charge is sufficiently high, charge regulation allows for large charge fluctuations. Interestingly, size polydispersity in charged colloids [27, 38–40] and neutral nanoparticles with hard-core repulsions [41–48] leads to glassy states. Here, charge polydispersity when Coulomb interactions are large leads to glassy states. In our model, we employ a random distribution for the charges on the surface of the nanoparticles. In a recent paper, the differences between a random and an ordered configuration of charges in a single particle was found to be small [49]. In a multiple particle system, a charge distribution of an ensemble of nanoparticles with ordered configurations may introduce quantitative differences to our results. However, we remain confident that the fundamental qualitative outcomes will continue when compared to the ordered arrangement of surface sites on the nanoparticle, which could be an interesting future study.

This work opens a large number of questions for further discussion, such as the effects of large concentrations of salt, multivalent ions and degree of charge polydispersity in binary solutions of particles that can take charge values of opposite sign or in macromolecules adsorbed to oppositely charged surfaces. One purpose of charge regulation is to model specific points where the dissociable sites are localized. In this sense, the model allows the interaction of these sites with the particles around it, including ions and nanoparticles. In previous work, theoretical expressions of the degree of dissociation as a function of attractive interactions and the density of the colloids have been derived [50]. Studying the behavior when the nanoparticles interact through attractive potentials via simulations would be important to verify theoretical results and to determine the degree of charge regulation in crowded environments resulting from short range attractions among nanoparticles, as well as adsorption to surfaces or interfaces. Furthermore, it is important to consider that in a constant pH environment, the migration of ions from the reservoir to the system is controlled by the simulation of chemical reactions within the grand canonical ensemble [13]. This requires the presence of a semi-permeable membrane that allows ions to pass through while preventing the passing of nanoparticles. In this context, ion migration between the system and the external reservoir is inherently integrated into our simulations. However, when charge regulation occurs in an isolated system, specifically within the canonical ensemble, disagreements emerge, particularly in scenarios involving high-volume fractions of nanoparticles and low salt concentrations as explained by Levin and Bakhshandeh [20]. Under these conditions, they found that the disparity in ion concentration within the system and the reservoir is attributed to an entropic contribution stemming from the Donnan Potential. It is important to note that while this discrepancy diminishes as salt concentration increases, a comprehensive exploration of the impact of the Donnan potential is indispensable in the context of pH-related investigations in canonical ensembles. This difference may introduce small variations in the phase diagram obtained in the canonical ensemble compared with the one obtained here. Further understanding of charge regulation and phase transitions in complex systems will allow us to engineer and tailor materials with enhanced properties and/or functionalities.

## Data Availability

The datasets generated and/or analyzed during the current study are available from the corresponding author on reasonable request.
